# Diagnosis and Management of Lemierre's Syndrome Presented with Multifocal Pneumonia and Cerebral Venous Sinus Thrombosis

**DOI:** 10.1155/2020/6396274

**Published:** 2020-03-22

**Authors:** Yasar Sattar, Ammu Thampi Susheela, Bibek Karki, Adnan Liaqat, Waqas Ullah, Fnu Zafrullah

**Affiliations:** ^1^Department of Internal Medicine, Icahn School of Medicine at Mount Sinai-Elmhurst Hospital, Queens, NY, USA; ^2^Department of Internal Medicine, Beth Israel Deaconess Medical Center, Boston, MA, USA; ^3^Department of Pulmonary and Critical Care, Mayo Clinic, Rochester, MN, USA; ^4^Department of Internal Medicine, Nexus Specialty Hospital, The Woodlands, TX, USA; ^5^Department of Internal Medicine, Abington Hospital Jefferson Health, Abington, PA, USA; ^6^Department of Internal Medicine, Steward Carney Hospital, Tufts Medical Center, Boston, MA, USA

## Abstract

A 27-year-old female patient initially presented with fever, myalgia, sore throat that progressed to multifocal pneumonia, and cerebral sinus venous thrombosis. A combination of upper respiratory symptoms with tooth infection, positive blood culture for *Fusobacterium nucleatum*, computed tomography (CT) chest finding of multifocal pneumonia, and magnetic resonance imaging (MRI) finding of internal jugular vein thrombosis (IJVT) and cerebral venous sinus thrombosis (CVST) suggested Lemierre syndrome. The patient was managed with fluids, antibiotics, and anticoagulants. The patient survived and discharged from the hospital. The patient's symptoms improved at 2 months of follow-up.

## 1. Introduction

Lemierre's syndrome is a rare disease, caused by the anaerobic bacteria *Fusobacterium*, most commonly *F. necrophorum* but occasionally *F. nucleatum*, *F. mortiferum*, and *F. varium* and is a rare fatal complication of acute oropharyngeal infection that can have up to 90% mortality [1]. Courmont and Cade reported the first case of Lemierre's syndrome in 1890. It affects 1 in a million population with 90% mortality [2]. It commonly affects adolescents or young adults of mean age 19–22 years with male (2 : 1) predominance [3, 4]. The case reports of Lemierre's syndrome are on the rise.

The source of primary infection can be in the chest, teeth, sinuses, and ears or trauma or head and neck malignancies. It manifests as metastatic septic emboli, with a tendency to rapidly involve organs such as lungs followed by liver, spleen, kidney, joints, soft tissues, and brain [3, 5]. It commonly presents with septic thrombophlebitis of the internal jugular vein and metastatic foci of infection [6]. The diagnosis of Lemierre's syndrome is based on the clinical presentation, a positive microbial culture, and a positive radiological finding.

## 2. Case Presentation

A 27-year-old female presented to the urgent care with 3 days of fever, generalized myalgia, and sore throat. At the urgent care, she did not receive antibiotics and was symptomatically managed. Her sore throat improved in a day but developed neck pain and hematuria that prompted her to visit emergency department (ED) after 2 days. She denied any other symptoms or any significant past medical history.

On general examination, she was alert, oriented to time, place, and person. She was febrile 101°F, stable blood pressure, stable heart rate, and SaO_2_ of 98% on room air. The patient had nonexudative erythematous pharynx and lateral neck tenderness. On auscultation, a systolic flow at the left upper sternal border and bilateral diffuse crackles in the lungs were heard. Splenomegaly was absent. Neurological examination was intact.

On admission, the laboratory study showed a white cell count of 21.6 × 10^3^ cells/microL with 89.5% neutrophils, positive left shift, platelet of 9 × 10^3^/microL, and hemoglobin of 10.0 g/dL with normal mean corpuscular volume. The reticulocyte count was normal, lactate dehydrogenase (LDH) was 210 U/L, and haptoglobin was 306 mg/dL. A peripheral smear showed normal red blood cell morphology, toxic granulation, and few giant platelets. Renal function showed increased blood urea nitrogen (BUN) 57 mg/dl, increased creatinine 3.43 mg/dl, and decreased eGFR 16 ml/min/1.73 m^2^ and sodium 131 mEq/l. Urinalysis showed gross hematuria with no RBC cast and negative nitrite/esterase. Arterial blood gas analysis revealed respiratory alkalosis with pH 7.482, pCO_2_ 30, normal bicarbonate, and anion gap.

Infectious workup includes blood culture positive for *Fusobacterium nucleatum* and negative rapid streptococcal test, throat culture, monospot test. Fecal leukocytes and ova/parasites tests were negative. A fungal workup was negative for histoplasma, blastomycosis, cryptococcus, and tuberculosis.

**Figure 1:**
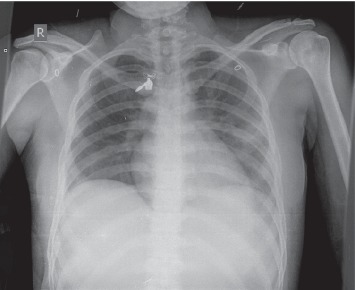
Chest radiography showing left lower and middle lobe pneumonia and a clear right lung.

An initial chest X-ray is shown in Figure 1.

**Figure 2:**
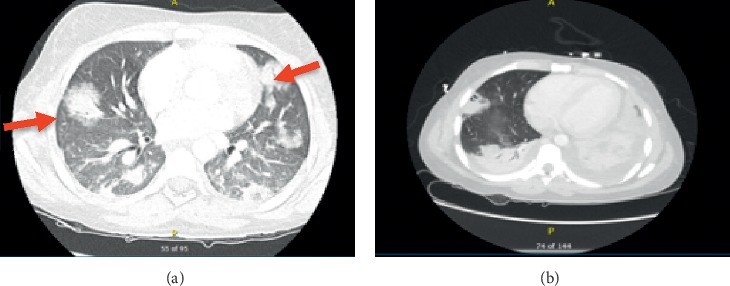
(a) High resolution CT chest showing septic nodules (red arrows) and bilateral pulmonary infiltrates. (b) CT chest showing bilateral pleural effusion with cavitary lesion likely septic thrombi largest of 4.4 cm.

**Figure 3:**
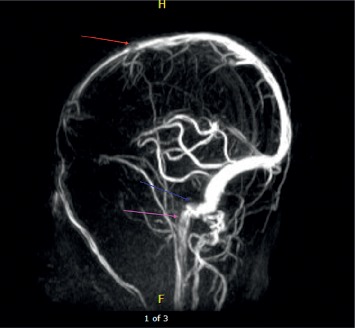
MRI venogram showing thrombus in the right sigmoid sinus (blue arrow), thrombus in the superior sagittal sinus (red arrow), and thrombus in the right jugular bulb (pink arrow).

Positive findings on chest CT and MRI venogram are shown in Figures 2 and 3, respectively. CT abdomen/pelvis and transthoracic echocardiography findings were normal.

**Figure 4:**
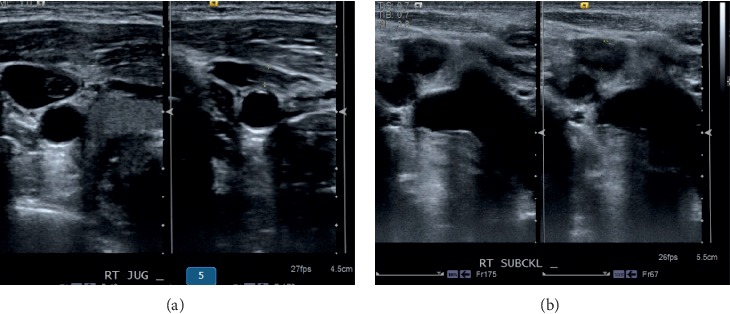
Venous duplex of the neck showing partially obstructed thrombus in the right internal jugular vein and right subclavian vein.

Venous imaging to see septic thrombophlebitis include upper extremity/neck venous duplex is shown in Figure 4.

Partial thromboplastin time was 26.4 seconds, D-dimer was 1223 ng/ml DDU, and fibrinogen was 752 mg/dL. The anticardiolipin antibody was positive, with an IgM level of 15.7 and low protein C with a level of 51 (normal 74–150%). An autoimmune workup for secondary causes of hypercoagulation was ruled out including lupus, factor-V Leiden mutation, and malignancy.

The patient was initially managed with fluids, broad-spectrum antibiotics such as meropenem and bridging mechanical ventilation in the intensive care unit. After imaging and blood culture were suggestive of Lemierre's syndrome, the patient was immediately started on piperacillin/tazobactam 4.5 gm IV 6 hourly and heparin drip with a goal aPTT of 60–80 seconds. The patient's symptoms significantly improved with this antibiotic regimen and anticoagulation. Heparin was switched to warfarin with a goal INR of 2–3, and piperacillin/tazobactam switched to clindamycin 300 mg q6hr with probiotic for 3 weeks for extended coverage of infection.

During the 2 months of follow-up, the patient had a complete resolution of symptoms, a positive beta-glycoprotein but normal IgM level. The patient was then scheduled for repeat MRI venogram in three months.

## 3. Discussion

The three classic criteria, i.e., a history of recent oropharyngeal infection, internal jugular venous thrombosis (IJVT) (clinical or radiological), and positive culture of *Fusobacterium nucleatum* should now be amended to include only the first two and start treating as presumed LS without waiting for the culture results which may delay diagnosis and treatment with increased risk of mortality.

In this case report, we presented a rare case of Lemierre's syndrome due to *Fusobacterium nucleatum* that can have potentially lethal consequences. Although there was no headache, which is also a classic presentation of the disease, the treating team was able to diagnose and provide timely treatment to the patient. Tenderness and stiffness on the lateral side of the neck along with a history of sore throat should elicit a high suspicion of Lemierre's disease. Hematuria in this patient is due to the metastatic septic emboli to the kidney. An accurate diagnosis by microbial cultures and imaging (CT/MRI and ultrasound) can facilitate aggressive treatments and favorable outcomes.

Once the oropharyngeal infection is established, *F. nucleatum* can penetrate through the facial planes or the lymphatic system to the nearby vasculature, potentially causing thrombosis as well as thrombophlebitis [7]. The internal jugular vein is usually involved due to the proximity to the peritonsillar space. In our case of Lemierre's disease caused by *F. nucleatum*, the right internal jugular vein, right transverse sinus, and sigmoid sinuses were involved. Our study involving *F. nucleatum* is the second case that showed the spread of the infection from the oropharynx into the right transverse sinus despite a relatively early diagnosis. As infection with *F. nucleatum* progresses extremely quickly, these patients should be treated with extreme caution. There are a few other aerobes, such as *E. coli*, *Streptococcus*, and *Staphylococcus* capable of causing Lemierre-like syndrome (LLS), which can also have very dangerous consequences [8].

Treatment of Lemierre's syndrome involves fluid resuscitation and prolonged antibiotic therapy. There have been reports that recannulation of the internal jugular vein or early use of anticoagulation can help with the penetration of antibiotics and increase the efficacy of the treatment [7, 9]. The choice of anticoagulant in Lemierre's syndrome has not been established due to its rarity. Prolonged use of anticoagulation can worsen prognosis as it can release septic emboli.

There have been only less than 160 case reports that have been published after its first reporting as a case series in 1936. There have been a handful of case reports published that have implicated *Fusobacterium nucleatum* as a cause of Lemierre's syndrome. After careful review of the several case reports, the differences in disease processes by *F. nucleatum* bacteria have been compiled in the following Table 1.

Although Lemierre initially denoted the case fatality to 90%, a case series by Riordan et al. reports the fatality between 0 and 18%. An average delay of 5 days from the time of admission to diagnosis has been reported. Almost 60–70% of cases require intensive care admissions [15, 16]. 37% of cases require intubation, and severe cases often require extracorporeal membrane oxygenation [17]. An average hospital stay of patients with Lemierre's syndrome is 3 weeks. Our case is a good addition to the existing pool of data about this forgotten disease.

## 4. Conclusion

Lemierre's syndrome presents initially with an oropharyngeal infection that causes septic thrombophlebitis of the neck veins and affects organs such as the lungs, liver, joints, kidney, and brain. It has potentially lethal consequences if intervention is delayed. Rapid and accurate diagnosis is done by early microbial culture, CT/MRI, and ultrasound, which facilitates aggressive treatment and a favorable outcome. The misdiagnosis or delayed diagnosis or faulty treatment increases the risk of mortality in this rare condition. Physicians of all specialties should have a low threshold for diagnosing Lemierre's syndrome in the light of distinctive signs and symptoms, including clinical, imaging, and blood cultures. Antibiotics are recognized as the primary mode of treatment. The use of anticoagulant in treating Lemierre's disease is debatable, and more prospective randomized trials are needed to come to a clear consensus.

## Figures and Tables

**Table 1:** Literature review of the reported cases on Lemierre syndrome with clinical presentation and management.

	Authors	Age/sex	Presentation	Thrombosis site	Diagnosis	Management	Outcomes	Follow-up	Ref
Case 1	Williams, et al.	19 y/M	Fever, rigors, sore throat, pleuritic chest pain, and productive cough with blood-tinged sputum, nausea, vomiting	Right external jugular vein (EJV)	Blood culture showed *Fusobacterium nucleatum*	Ampicillin-sulbactam, heparin	Complete resolution of symptoms on day 5	Patient did not follow-up	[10]

Case 2	Takahashi, et al.	73 y/M	Fever, occipital pain, diplopia, and right ptosis	Left internal jugular vein	Blood culture showed *Fusobacterium nucleatum*	Antibiotics	Complete resolution of symptoms	Not mentioned	[11]

Case 3	Wong, et al.	21 y/F	Sore throat, nausea, vomiting, and fever, swollen neck glands, right ear pain, dysphagia, dry cough, palpitations, back pain, and orange urine discoloration	Internal jugular vein	Blood culture showed *Fusobacterium nucleatum*	Antibiotics, metronidazole, enoxaparin, warfarin	Complete resolution of symptoms	Continued improvement	[12]

Case 5	Cheung, et al.	24 y/M	Fever, neck pain, rhinorrhea, sore throat, and tender cervical lymph nodes	Hepatic vein thrombosis	Blood culture showed *Fusobacterium nucleatum*	Antibiotics, anticoagulants	Resolution of symptoms	Not mentioned	[13]

Case 6	Iwata, et al.	23 y/M	Fever, sore throat, neck pain, and chest pain	Right internal jugular vein	BAL culture showed *Fusobacterium nucleatum*	Antibiotics	Resolution of symptoms	Not mentioned	[14]

BAL: Bronchoalveolar lavage.
